# The Prevalence of Anxiety and Depression in Children With Postural Orthostatic Tachycardia Syndrome (POTS): A Retrospective Study

**DOI:** 10.7759/cureus.69941

**Published:** 2024-09-22

**Authors:** Bahram Kakavand, Aliya Centner, Safia Centner, Shirin Hasan

**Affiliations:** 1 Pediatric Cardiology, Nemours Children's Health System, Orlando, USA; 2 Medicine, University of Central Florida College of Medicine, Orlando, USA; 3 Child and Adolescent Psychiatry and Pediatrics, Nemours Children's Hospital, Orlando, USA

**Keywords:** gad-7 score, nine-item patient health questionnaire (phq-9), depression, generalized anxiety disorder (gad), postural orthostatic tachycardia syndrome (pots)

## Abstract

Introduction: Postural orthostatic tachycardia syndrome (POTS) is a chronic form of orthostatic intolerance characterized by various symptoms such as dizziness, lightheadedness, and increased heart rate. Conflicting reports exist regarding the prevalence of anxiety and depression in adults with POTS, while data on pediatric POTS remains scarce.

Method: A retrospective analysis of pediatric patients aged 11-17 years with POTS, who underwent autonomic testing at Nemours Children’s Hospital in Orlando, Florida, was conducted. The patients were screened for anxiety, using the Severity Measure for Generalized Anxiety Disorder-Child Age 11-17 years (GAD-7) questionnaire, and depression, using PHQ-9 Modified for Adolescence (PHQ-A) for depression. The prevalence rates of anxiety and depression in the study cohort were compared to historical data from similar age groups in the existing literature. The study was approved by the Nemours Children’s Hospital Institutional Review Board.

Results: The cohort comprised 27 children with POTS (26 females, age 15.8±1.6 years). Overall, 74% exhibited moderate-to-severe anxiety, depression, or both, with 44% having comorbid anxiety and depression. In total, 4/27 (14%) had pure depression and 4/27 (14%) had pure anxiety. Six patients had no depression or anxiety. On average, POTS symptoms began 1.9±1.3 years before diagnosis. Eleven patients took stable doses of psychotropic medications. After a follow-up period of 5.1±1.7 months of POTS therapy, seven patients had follow-up questionnaires. In 4/7 patients, the depression severity improved, and in 3/7 patients, the anxiety severity improved. Patients were not actively treated for depression and anxiety during this time.

Conclusion: Anxiety and depression are prevalent among pediatric patients with POTS. While preliminary data suggests POTS therapy may alleviate these psychological symptoms, further longitudinal studies are warranted to explore the therapeutic impact in greater detail.

## Introduction

Postural orthostatic tachycardia syndrome (POTS) is a chronic form of orthostatic intolerance characterized by an excessive heart rate increase upon assuming an upright position, leading to a range of symptoms including lightheadedness and dizziness [1]. POTS is diagnosed when there is a heart rate increase of at least 30 beats per minute (bpm) within 10 minutes of standing in adults or 40 bpm in children without corresponding hypotension on the head-up tilt table [1]. In patients less than 19 years of age, the threshold is higher due to age-related physiological differences [1].

POTS is a common form of orthostatic intolerance, affecting between 0.2% and 1% of the adolescent and adult population in developed countries [2]. The majority of POTS patients are typically young females aged 15-45 years, with studies suggesting a higher prevalence among Caucasians [2].

Patients with this diagnosis often present with lightheadedness, dizziness, palpitations, tremulousness, fatigue, headaches, nausea, chest pain, and difficulty breathing [1]. Many of these patients may also experience presyncopal episodes, though syncope itself is not major, defining characteristic [1]. It is estimated that around one-fourth of POTS patients are disabled and are not able to partake in regular activities [1].

Many patients with POTS may present with comorbid conditions, including but not limited to Ehlers-Danlos syndrome (EDS), mast cell activation, and autoimmune conditions [3]. The estimated frequencies may be variable among populations, and thorough evaluations have not been conducted among a large POTS population [3]. Studies have estimated that around 40% of POTS patients have migraines, 20-30% meet diagnostic criteria for EDS, and 15% for autoimmune disease diagnoses [3].

Patients with POTS frequently experience a reduced quality of life, with many reporting co-occurring anxiety and depression, further complicating symptom management. Many patients may receive the diagnosis of an anxiety or depressive disorder prior to being diagnosed with POTS [1]. While POTS patients are commonly perceived to be anxious, some studies have indicated that this is due to the intersection between common anxiety symptoms and orthostatic symptoms [1]. Further studies in adults suggest POTS patients are more anxious than their peers without POTS but are significantly less anxious than patients with the diagnosis of panic disorder [2]. There is also consistent evidence of depression in POTS, with most studies indicating that its severity is generally mild-to-moderate [1]. Several studies have also found a positive correlation between the intensity of self-reported orthostatic symptoms and the level of depression [1].

Determining whether anxiety and depression are distinct comorbidities or manifestations of POTS remains a critical area of ongoing research, with significant implications for diagnosis and treatment strategies [1]. However, it remains clinically important to consider the possibility of a comorbid anxiety and/or depressive disorder in patients with POTS [1].

Generalized anxiety disorder (GAD) can be found in 2.2% of adolescents [4]. Patients with GAD are frequently over worried and bothered about health, family, work, assets and several other aspects of everyday life [5]. This constant worry often interferes with the individual's ability to function [5]. Patients may present with symptoms of restlessness, tiredness, irritability, tremulousness, worry, irritability, and sweating, and for a formal diagnosis of GAD, these symptoms must be constant, persistent, and pervasive for at least six months [5]. Patients can be screened in a clinical setting using the Generalized Anxiety Disorder scale, often referred to as GAD-7, which can be used as a metric to assess a patient’s anxiety level [6]. The GAD-7 presents a list of problems and feelings and asks patients to quantify how often these symptoms are experienced, and based on their response, anxiety levels can be classified as minimal, mild, moderate, or severe [7].

Major depressive disorder (MDD) is diagnosed when an individual experiences a consistently low or depressed mood, diminished interest in previously enjoyable activities (anhedonia), feelings of guilt or worthlessness, low energy, difficulty concentrating, changes in appetite, psychomotor slowing or agitation, sleep disturbances, or suicidal thoughts [8]. Similar to the GAD-7, the Patient Health Questionnaire-9 (PHQ-9) is a tool used to classify patient’s symptoms of depression [9]. The levels can be classified as minimal, mild, moderate, moderately severe, or severe [10]. In a retrospective study, 18 of 144 adolescents (12.5%) were found to have moderate-to-severe depression using the PHQ-9 questionnaire [11].

Data on anxiety and depression in children with POTS is scant. This retrospective study aimed to determine the prevalence of anxiety and depression in pediatric patients diagnosed with POTS and assess whether POTS treatment influences these psychological symptoms.

This article was previously presented as a poster at the 2019 American Autonomic Society Annual Meeting in Clearwater Beach, Florida, on November 7, 2019.

## Materials and methods

The retrospective cross-sectional study was approved by the Nemours Children’s Hospital Institutional Review Board, ensuring compliance with all relevant ethical standards for research involving human participants. We retrospectively reviewed the electronic medical records of pediatric patients aged 11-17 years who were diagnosed with POTS and treated at the outpatient cardiology clinic of Nemours Children’s Hospital, Orlando, prior to April 29, 2019.

As part of standard care, patients are administered screening questionnaires for anxiety and depression during their initial evaluation. If the patient is found to have anxiety and/or depression, they are provided with resources to contact a mental health provider.

Patients who were already receiving care from a mental health provider for anxiety and/or depression were excluded from receiving the screening questionnaire and considered to have moderate-to-severe anxiety and/or depression. A subset of patients completed follow-up questionnaires approximately three to six months after the initiation of POTS therapy to assess changes in anxiety and depression symptoms. A total of 27 patients were found to have adequately and accurately recorded data and were included in the study. Patients were diagnosed with POTS based on autonomic testing, applying the criteria of a 40 bpm increase in heart rate during a head-up tilt table test without concurrent hypotension, as per the published diagnostic guidelines for pediatric populations [1].

**Figure 1:**
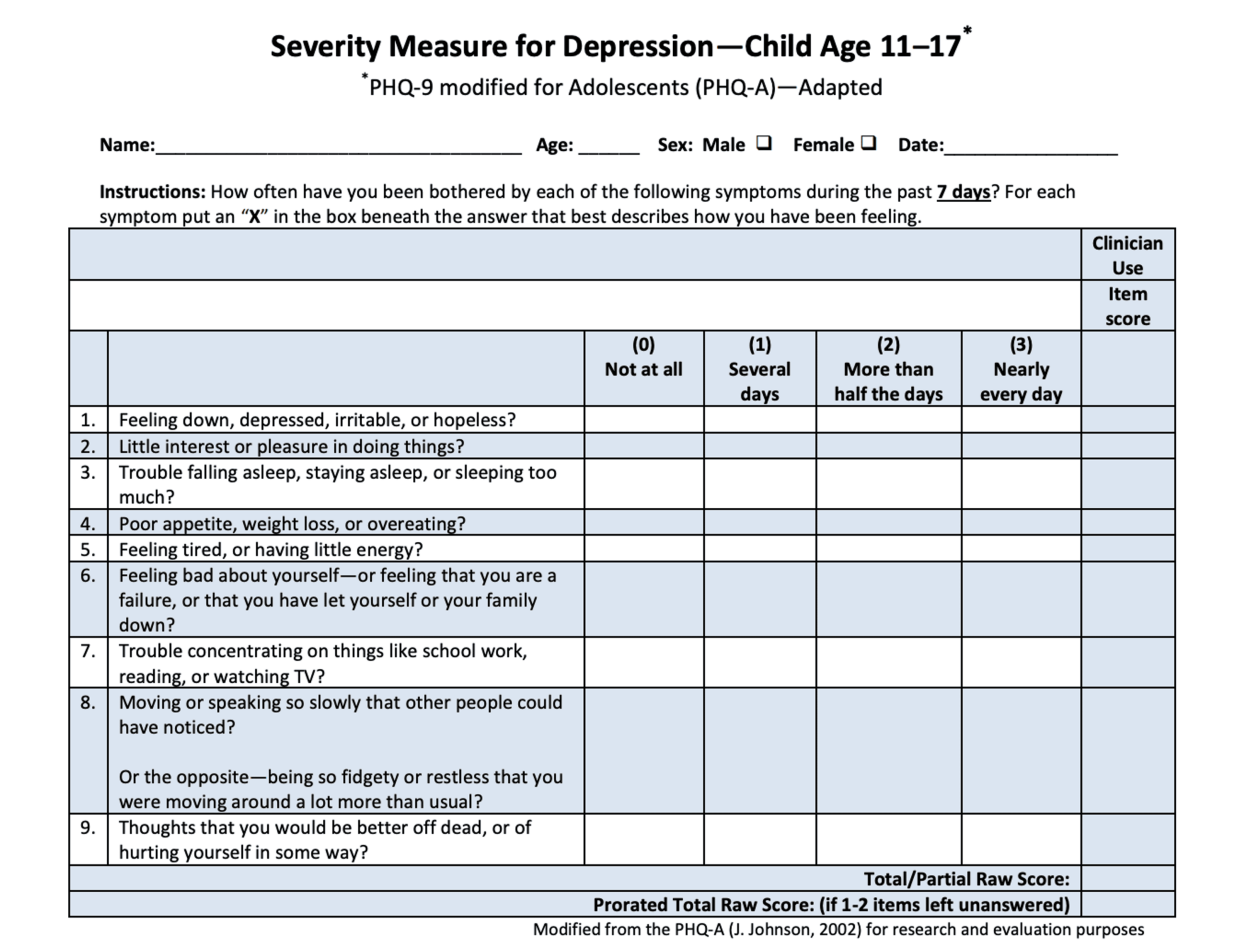
Patient Health Questionnaire-9 (PHQ-9) modifies for adolescents (PHQ-A) screening questionnaire for depression in adolescents. Severity Measure for Depression-Child Age 11-17 years, this measure was adapted from the PHQ-9 modified for adolescents (PHQ-A), which is in the public domain.

**Figure 2:**
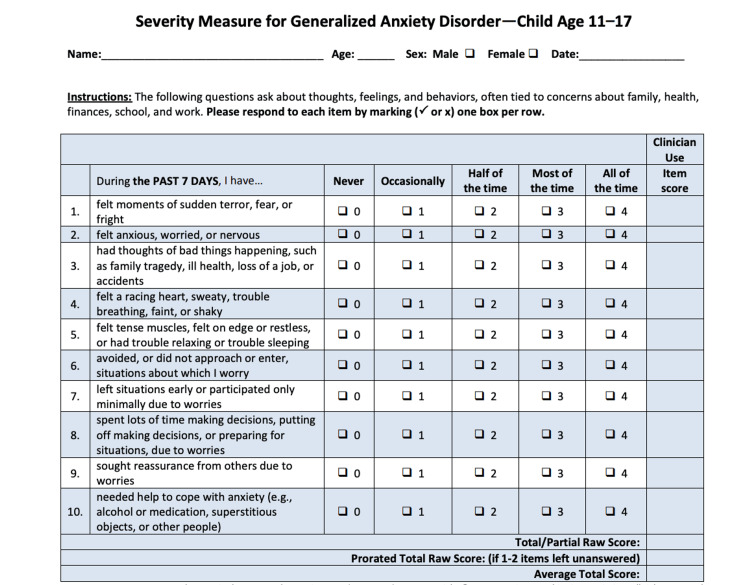
Severity Measure for Generalized Anxiety Disorder-Child Age 11-17 years (GAD-7) screening questionnaire for anxiety in adolescents. Severity Measure for Generalized Anxiety Disorder-Child Age 11-17 years, this measure is adapted from Craske et al. (2013), copyrighted by the American Psychiatric Association, and can be reproduced without permission by researchers and by clinicians for use with their patients.

Validated screening tools, including the Patient Health Questionnaire-9 modified for adolescents (PHQ-A) for depression (Figure 1) and the Severity Measure for Generalized Anxiety Disorder-Child Age 11-17 years (GAD-7) were administered (Figure 2). Descriptive statistics were used to describe patients’ demographics and determine the prevalence of anxiety and depression in this population.

## Results

The study included 27 pediatric patients diagnosed with POTS, of which 26 were female. The mean age was 15.8±1.6 years. On average, POTS symptoms began 1.9±1.3 years before diagnosis. Six patients (22%) exhibited no signs of anxiety or depression, a finding that contrasts with the high prevalence reported in other studies. As shown in Table 1, of the 27 patients, 20 (74%) exhibited moderate-to-severe anxiety, depression, or both. Specifically, four (14%) had only depression, four (14%) had only anxiety, and 12 (44%) experienced both conditions.

**Table 1:** Number of patients with moderate-to-severe anxiety and/or depression.

Variables	Number of patients
Moderate-to-severe anxiety (only)	4
Moderate-to-severe depression (only)	4
Both moderate-to-severe anxiety and depression	12

Twenty-two patients took the PHQ-9, a depression screening questionnaire for adolescents. Of these patients, six had no depression, three had mild depression, six had moderate depression, five had moderately severe depression, and two had severe depression (Table 2). As shown in Table 3, five patients were already seeing mental health providers and therefore were not given the PHQ-9. Three were categorized as moderate-to-severe and two had no depression but were seen by mental health providers for other reasons.

**Table 2:** Depression severity based on questionnaire.

Depression severity (based on questionnaire)	Number of patients
None	6
Mild	3
Moderate	6
Moderately severe	5
Severe	2

**Table 3:** Depression severity (did not take questionnaire but sees mental health provider). Patients who were already receiving care from a mental health provider for anxiety and/or depression did not receive the screening questionnaire as they already had a diagnosis. If they had a diagnosis of depression from the mental health provider, they were considered to have moderate-to-severe depression.

Depression severity (did not take questionnaire but sees mental health provider)	Number of patients
None	2
Moderate-to-severe	3

Twenty-two patients completed the GAD-7, an anxiety screening questionnaire for adolescents. Of these patients seven had no anxiety, three had mild anxiety, five had moderate anxiety, six had severe anxiety, and one had extreme anxiety (Table 4). As shown in Table 5, five patients were already seeing mental health providers and therefore were not given the GAD-7. Four were categorized as moderate-to-severe and one had no anxiety but was seen by mental health providers for other reasons.

**Table 4:** Anxiety severity (based on questionnaire).

Anxiety severity (based on questionnaire)	Number of patients
None	7
Mild	3
Moderate	5
Severe	6
Extreme	1

**Table 5:** Anxiety severity (did not take questionnaire but sees mental health provider). Patients who were already receiving care from a mental health provider for anxiety and/or depression did not receive the screening questionnaire as they already had a diagnosis. If they had a diagnosis of anxiety from the mental health provider, they were considered to have moderate-to-severe anxiety.

Anxiety severity (did not take questionnaire but sees mental health provider)	Number of patients
None	1
Moderate-to-severe	4

**Figure 3:**
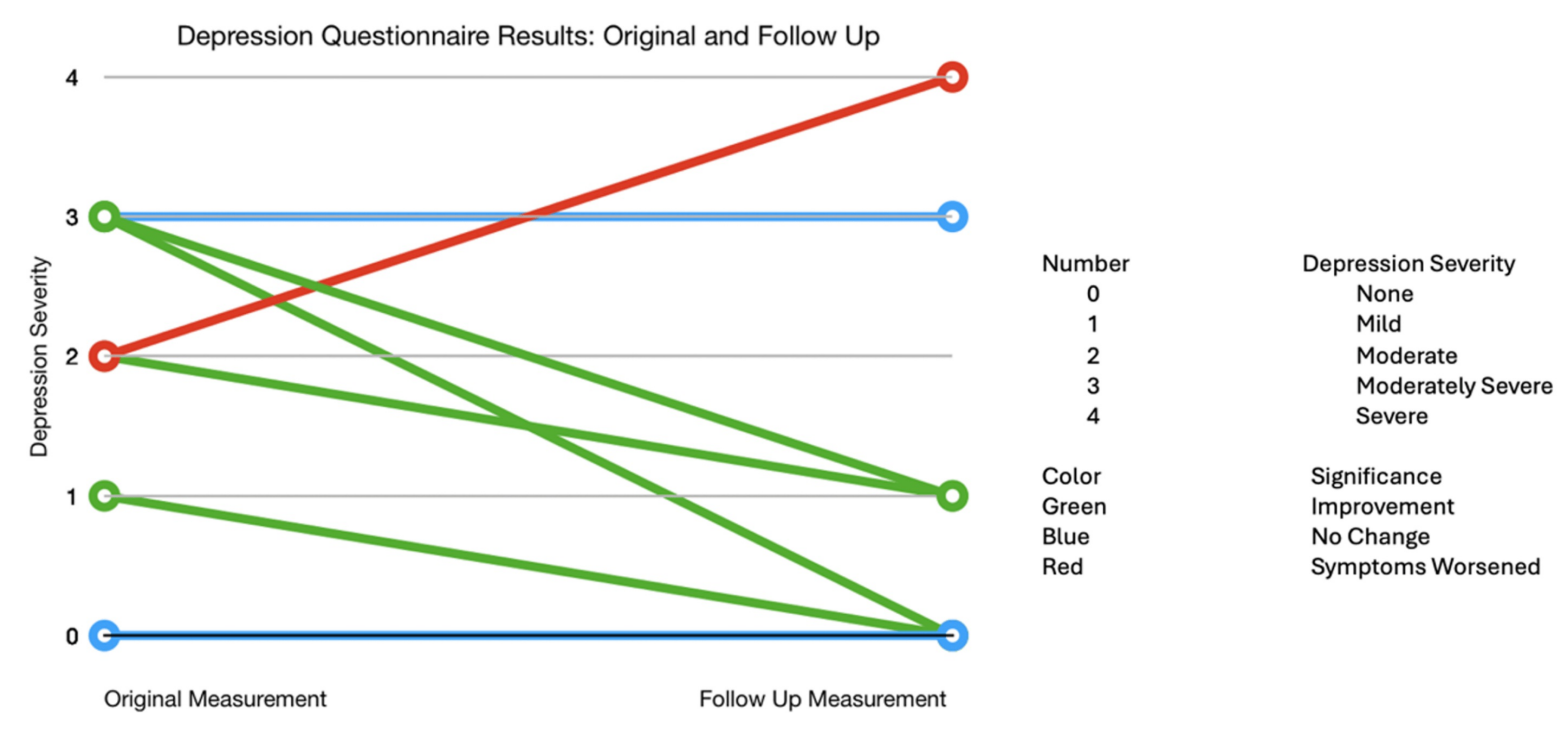
Depression questionnaire results: original and follow-up.

**Figure 4:**
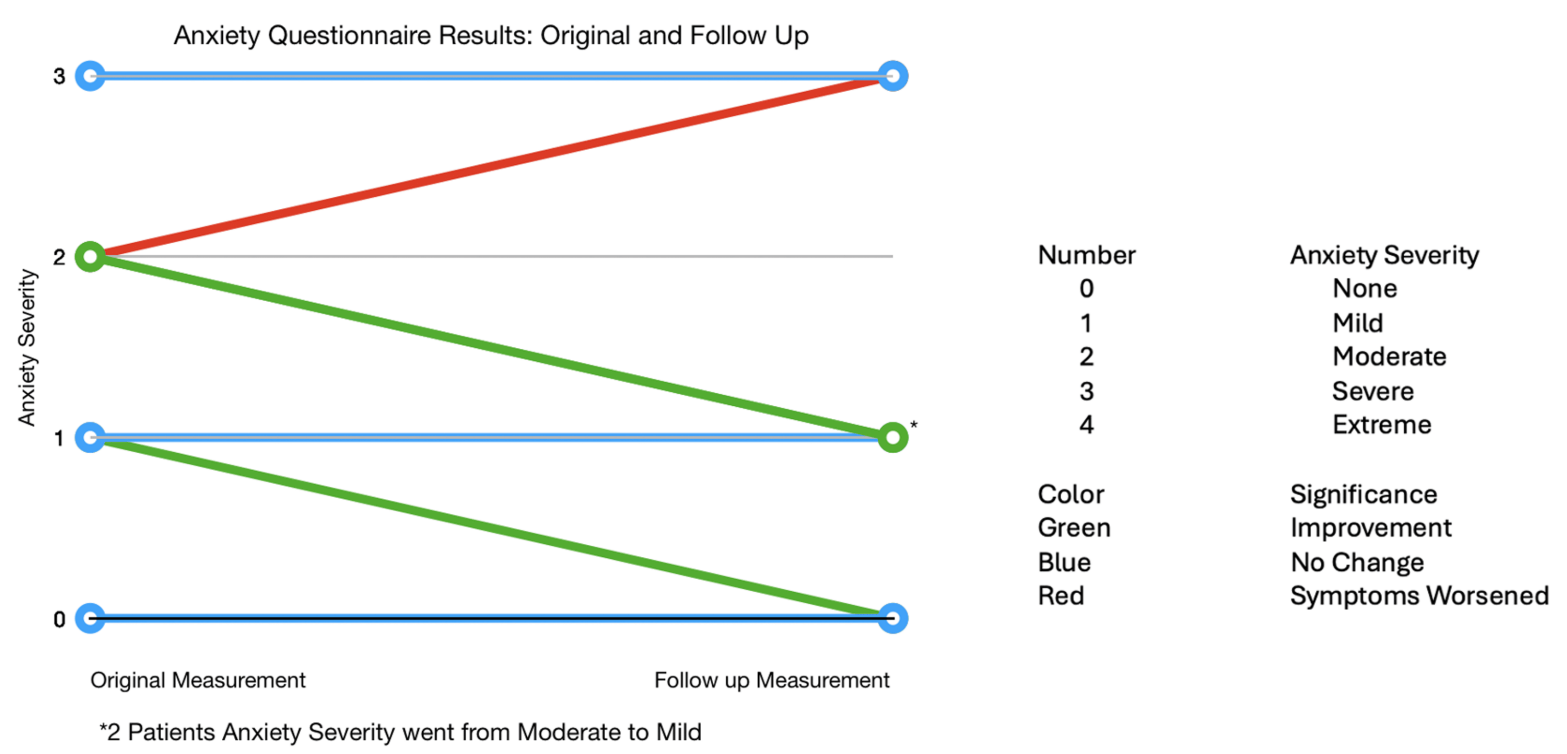
Anxiety questionnaire results: original and follow-up.

Eleven patients took stable doses of psychotropic medications. Following a mean period of 5.1±1.7 months of POTS therapy, follow-up questionnaires were administered to seven patients to assess changes in anxiety and depression. Among the seven patients with follow-up data, four (57%) showed a reduction in depression severity, while three (43%) demonstrated improvement in anxiety symptoms (Figures 3, 4).

## Discussion

Physiological symptoms, particularly anxiety and depression, are frequently reported in patients with POTS, though the mechanisms linking autonomic dysfunction and these mental health conditions remain the subject of ongoing research [12]. POTS, a condition characterized by an abnormal increase in heart rate upon standing, significantly impacts various aspects of a patient's life, including their mental health [1]. Research suggests that the psychological distress experienced by POTS patients, particularly anxiety and depression, may be exacerbated by the autonomic dysfunction inherent to the condition, though further studies are needed to establish causality.

Studies have demonstrated a notable association between POTS and depressive disorders as well as anxiety, particularly within the adult population. Studies in adults indicate that 87% of individuals with POTS experienced mild-to-moderate depressive symptoms; similar trends are observed in pediatric populations, though comprehensive data remain limited [13]. Additionally, over half of these patients report disturbances in sleep patterns, and many experience symptoms of mild-to-moderate anxiety [14]. Psychological symptoms such as anxiety and depression can intensify the physical manifestations of POTS, such as palpitations and fatigue, creating a feedback loop that complicates both the diagnosis and management of the condition [13].

In the context of pediatric POTS patients, research on the correlation between POTS and psychological symptoms, such as depression and anxiety, is comparatively limited. Nevertheless, some studies have highlighted that adolescents with POTS encounter significant functional impairments and psychological distress [14]. This suggests that the impact of POTS on mental health in younger populations may be considerable, though further research is needed to fully understand the scope of these effects [14].

In both adults and adolescents, the impairments caused by POTS can be extensive. Adult patients often face challenges in performing routine daily activities, including work, physical exercise, and even basic self-care tasks such as eating and showering [14]. Similar difficulties have been observed in pediatric patients, who also may struggle with activities critical to their development and social interaction [14]. The resulting psychological burden often manifests as anxiety and depression, exacerbating the overall impact of the syndrome. Patients with POTS frequently report symptoms such as fatigue, sleep difficulties, and a diminished quality of life, which are closely intertwined with their psychological well-being [15].

Although POTS can severely limit daily functioning and overall quality of life, it is often responsive to both conservative management strategies and pharmacological treatments [16]. Treatment approaches for POTS, which may include lifestyle modifications, dietary adjustments, physical therapy, and medications, have shown promise in alleviating some of the associated psychological symptoms. Evidence suggests that effective management of POTS may also lead to improvements in anxiety and depression, although the degree of improvement can vary among individuals [16].

In our study, it was found that 16 out of 27 (59.3%) patients reported moderate-to-extreme anxiety, and 16 out of 27 (59.3%) also reported moderate-to-severe depression. In comparison, the prevalence of anxiety and depression among adolescents in general populations is much lower, with rates of 2.2% and 12.5%, respectively [4,11]. Post-treatment observations revealed that four out of seven patients showed improvement in depressive symptoms, while three out of seven experienced reduced anxiety symptoms. Conversely, one out of seven patients reported worsened depressive symptoms, and another one out of seven had exacerbated anxiety symptoms. These findings suggest that POTS treatment may offer some relief from anxiety and depression, although individual responses can vary.

Several limitations should be considered when interpreting these findings. The study in question was retrospective and involved a relatively small sample size, which may not be representative of the broader population of POTS patients. Furthermore, only a limited proportion of patients received follow-up assessments to evaluate the long-term impact of treatment on mood symptoms. Additionally, the study was conducted at a single center, which may limit the generalizability of the results. Future research with larger, more diverse samples and prospective study designs is needed to better understand the relationship between POTS and psychological symptoms and to refine treatment approaches to address both physical and psychological aspects of the syndrome.

## Conclusions

Anxiety and depression are significantly more prevalent in pediatric patients with POTS compared to the general adolescent population, highlighting the need for integrated mental health evaluation in this group. The co-occurrence of anxiety and depression can complicate the management of POTS, particularly by reducing the efficacy of standard therapies and requiring additional mental health interventions. Pediatric POTS patients should routinely be screened for anxiety and depression. Effective treatment of POTS may lead to improvements in anxiety and depression symptoms, though further research is required to explore the extent of this relationship and identify optimal therapeutic strategies.
